# Effects of clinical clerkship in education for physical and occupational therapy students: a multifaceted examination using objective indices

**DOI:** 10.20407/fmj.2019-024

**Published:** 2020-03-25

**Authors:** Nozomi Odo, Kei Ohtsuka, Yukari Suzuki, Fumihiro Matsuda, Soichiro Koyama, Tetsuro Watari, Hiroaki Sakurai, Norikazu Nakagawa, Yoshikiyo Kanada

**Affiliations:** 1 Faculty of Rehabilitation, School of Health Sciences, Fujita Health University, Toyoake, Aichi, Japan; 2 Masuhara Clinic, Osaka, Osaka, Japan

**Keywords:** Clinical clerkship, Stress, Sleep, Training evaluation

## Abstract

**Objectives:**

This study aimed to determine the effects of clinical clerkship in physical and occupational therapy students’ education on their stress, sleep, and technical skill acquisition.

**Methods:**

We compared responses to the Brief Job Stress Questionnaire and the Athens Insomnia Scale, and students’ clinical training grades between a traditional clinical training group (n=48) and a clinical clerkship group (n=48).

**Results:**

Compared with the traditional group, the clinical clerkship group showed significantly higher scores on the Brief Job Stress Questionnaire for quantitative and qualitative burden, and significantly lower scores for the extent of control over tasks, irritability, fatigue, depression, and physical ailment. Scores for vitality and supervisor support were also significantly higher in the clinical clerkship group than the traditional group. The median Athens Insomnia Scale score was significantly lower in the clinical clerkship group. Clinical training grades for fundamental attitude and treatment techniques were significantly higher in the clinical clerkship group than in the traditional group.

**Conclusions:**

Students that experienced clinical clerkship perceived quantitative and qualitative burdens, which may be attributable to the level of interaction with patients during training. Their perception of low control over tasks may be because their supervisors described tasks specifically. However, the clinical clerkship group showed lower mental and physical stress than the traditional group. These students perceived they had supervisor support, which may be attributable to increased communication with their supervisor. Clinical clerkship was also linked to better sleep status than traditional training. Continuing clinical clerkship is necessary to develop students’ technical clinical skills.

## Introduction

In Japan, clinical training of physical and occupational therapists (hereinafter referred to as “therapists”) consists of an assigned-patient training format in which students are assigned a few patients to evaluate and treat under supervision. This format is used in many training schools. Aside from the practical training involving assigned patients, students spend most of their time observing, and therefore have insufficient practical experience. This experience can only be gained with patients in the clinical setting, meaning the assigned-patient training format has been viewed as problematic. Another issue is that students spend many hours writing case reports regarding their assigned patient(s) or performing other tasks after finishing their clinical training time. This is because instructions on required case reports are often given after clinical work is completed. In 2017, the Study Group for Improving the Physical Therapist and Occupational Therapist Training School Curriculum conducted a survey of current students (n=414) and graduates (n=1244) of physical and occupational therapy programs.^1^ The survey found that approximately 80% of students could not finish tasks within their clinical training time and took work home each day; 64.3% of physical therapy students and 73.5% of occupational therapy students spent at least 3 hours a day at home on these tasks. Moreover, the survey showed that approximately 60% of students slept 3–5 hours less on clinical training days than on lecture days, and approximately 50% felt mentally and physically unwell. Clinical training often demands difficult technical skills from students who may have inadequate skills and are in an unfamiliar environment. This means students may be in a continuous nervous state and frequently experience insomnia and stress. Many training schools consider insomnia^2^ and stress^3^ among clinical trainees as problematic. The National Diet assembly also debated about suitable clinical training approaches for therapists in 2016.^1^

To resolve such issues in the clinical training of therapists, the Japan Ministry of Health, Labour and Welfare (MHLW) issued the “Teaching Rules and Guidelines for Physical and Occupational Therapist Training Schools” in October 2018. These guidelines specify clinical clerkship as the recommended format for clinical training. Clinical clerkship refers to students’ proactive participation in clinical care within a clinical team comprising students and supervisors.^4–6^ In this training method, students learn their professional identity (knowledge, skill, sense, emotion by assisting in physical/occupational therapy practice and gaining a variety of experience based on their supervisor’s advice and instruction.^7,8^

At the Fujita Health University Faculty of Rehabilitation, School of Health Sciences (hereinafter referred to as “our university”) in which the present authors work, students receive 1,590 hours of clinical training. This amount is approximately 1.96-fold greater than the 810 hours specified as a part of the Clinical-Oriented System for Progression & Innovation of Rehabilitation Education (COSPIRE) project. Clinical training consists of initial hands-on training during the second year (3 weeks at the university hospital), clinical training during the third year that comprises three 7-week sessions at the university hospital, and applied practical training during the fourth year comprising two 6-week sessions at an external institution. In the initial hands-on training and third-year clinical training, university teachers engage in clinical practice and work together with university hospital supervisors to instruct students.

In the 2017 academic year, our university switched from assigned-patient training to clinical clerkship for the initial hands-on training and third-year clinical training at the university hospital. In clinical clerkships at our university, students are not assigned specific patients; rather, students participate in all patient care for which their supervisor is responsible in accordance with that student’s learning progress and the level of technical difficulty. This is the fundamental principle of the clinical clerkship.

Student participation steps involve “observing,” “imitating,” and “practicing with the supervisor.” In the “observing” phase, students observe the techniques performed by their supervisor while the supervisor explains the process in such a way that the student is not a mere spectator. In the “imitating” phase, students repeat the observed technique several times with the guidance and support of their supervisor. Finally, in the “practicing with the supervisor” phase, students perform the imitation technique under the supervision of their supervisor. After each step, the student’s level of understanding and skill acquisition are verified, and appropriate feedback is given. A checklist of goals and technical skills is created, and the supervisor confirms the progress made with the student. Because the training consists of learning skills in a clinical setting, formal written reports are not required, and important records are summarized in the student’s daily notebook. The supervisor’s instructions to their student are generally completed within work hours, and time is allotted within these hours for students to write summaries in their daily notebook and reflect on the day’s clinical training.

The present study aimed to validate the effects of clinical clerkship, which was introduced in our university in the 2017 academic year, by comparing stress status, sleep status, and skill acquisition during clinical training between a traditional clinical training group and clinical clerkship group from a multifaceted perspective.

## Methods

This study included 96 physical or occupational therapy students from our university. This included 48 students who had experienced clinical training in the 2016 academic year before the introduction of clinical clerkship; these students were classified as the “traditional group.” Forty-eight students were randomly selected from the 98 students who had experienced clinical clerkship during their third-year clinical training in the 2017 academic year, and classified as the “clinical clerkship group.”

We used the Brief Job Stress Questionnaire created by the MHLW to evaluate students’ stress status, the Japanese version of the Athens Insomnia Scale to evaluate their sleep status, and clinical training grades from our university to evaluate skill acquisition. The Brief Job Stress Questionnaire^9^ includes 18 items with 55 questions. Nine items cover job stress factors, six items cover stress response, and three items cover social support. Items related to job stress factors investigate causes of stress at work (“psychological quantitative burden of the job,” “psychological qualitative burden of the job,” “subjective feeling of physical burden,” “interpersonal stress,” and “environmental stress”); a higher score corresponds to a higher level of stress. For “extent of control over the job,” “extent of using technical skills,” “job suitability,” and “rewarding,” a lower score corresponds to a higher level of stress. Items related to stress response indicate a response induced by stress. For “irritability,” “fatigue,” “anxiety,” “depression,” and “physical ailment,” a higher score corresponds to a greater level of stress. For items covering “vitality,” a lower score corresponds to a higher level of stress. Items related to social support indicate how much support the respondent perceives (“support from superiors,” “support from co-workers,” and “support from friends and family”); a lower score corresponds to a higher level of stress. Questions for these items are answered on a four-point scale (1=very much, 2=moderately so; 3=somewhat; 4=not at all), and can monitor stress in the past month. This questionnaire has been used in job stress surveys involving laborers as well as with healthcare providers such as residents^10^ and therapists.^11^ The reliability and validity of the questionnaire have been reported.^9^ In the present study, we replaced the term “job” with “clinical training” or “task,” “superior” with “supervisor,” and “co-worker” with “teacher.”

The Athens Insomnia Scale was developed by the World Health Organization, and assesses insomnia using questions on daytime and nighttime sleep during the past month through a point-addition system. The reliability and validity of the scale have been reported by Constantin et al.^12^ In the present study, we used the Japanese version of this scale developed by Okajima et al.^13^ The Japanese version of the Athens Insomnia Scale comprises eight questions, giving a total possible score of 24; a score of 4 or 5 corresponds to suspected insomnia, and a score of ≥6 corresponds to a high probability of insomnia.

Clinical training items from our university (Table 1) were created based on the “Handbook of Clinical Training Education (third edition)” by the Japanese Physical Therapy Association. This covers three skill areas: fundamental attitude (11 items; score of 55), evaluation technique (14 items; score of 75), and treatment technique (10 items; score of 50). There are 35 items in total. Each item is evaluated from 0 to 5, giving a total possible score of 175. The grade is determined by the supervisor on discussion with staff and teachers in the supervisor’s clinical group. This grade table was used in the present study to compare grades between the traditional group and the clinical clerkship group (i.e., before and after the introduction of clinical clerkship).

Students in both groups responded to the Brief Job Stress Questionnaire and the Japanese version of the Athens Insomnia Scale anonymously at the end of their fourth-year clinical training. The responses were compared between the two groups. In addition, the total score for the three skill areas and the score for each skill on the clinical training grades during the first session of the third-year clinical training were compared between the two groups. We used the Wilcoxon rank-sum test for the statistical analyses. P<0.05 was considered statistically significant. All analyses were performed with IBM SPSS 21.0.

The present study was approved by the Institutional Review Committee of Epidemiological and Clinical Research of Fujita Health University (HM17-010, HM17-144). All data were managed using unlinkable anonymization and were treated with care to protect students’ privacy.

## Results

The response rate for the Brief Job Stress Questionnaire was 100% for both groups. The response rate for the Japanese version of the Athens Insomnia Scale was 91.7% (44/48) in the traditional group and 100% in the clinical clerkship group.

Table 2 shows the median, mean, and results of the statistical analysis for the Brief Job Stress Questionnaire items. The scores for the psychological quantitative burden of the task (quantitative burden) and the psychological qualitative burden of the task (qualitative burden) were significantly higher in the clinical clerkship group compared with the traditional group. Conversely, the scores for the extent of control over tasks were significantly lower in the clinical clerkship group compared with the traditional group. Analysis of the stress response items showed that compared with the traditional group, the clinical clerkship group had significantly lower scores for irritability, fatigue, depression, and physical ailment and significantly higher vitality scores. For social support, the clinical clerkship group had significantly higher scores on training supervisor support compared with the traditional group.

**Figure 1:**
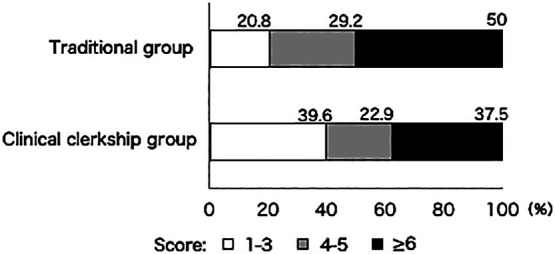
Comparison of sleep status between the traditional and clinical clerkship groups (Japanese version of the Athens Insomnia Scale). a. Percentages of different scores. The percentages of different scores in the traditional and the clinical clerkship groups are shown. In the clinical clerkship group, the percentages of those with a score of 4 or 5 and ≥6 were lower compared with the traditional group. b. Comparison of median and mean. The median was significantly lower in the clinical clerkship group compared with the traditional group.

Figure 1 shows the results of the Japanese version of the Athens Insomnia Scale. In the traditional group, 20.8% had a score of 1–3 (no problem), 29.2% had a score of 4 or 5 (suspected insomnia), and 50% had a score of ≥6 (high probability of insomnia). In contrast, 39.6% of the clinical clerkship group had a score of 1–3, 22.9% had a score of 4 or 5, and 37.5% had a score of ≥6. This indicated that the percentage of students with a score of 4 or 5 or ≥6 was lower after the introduction of clinical training. Moreover, the median score was significantly lower in the clinical clerkship group (4) than in the traditional group (5.5) (Table 3).

**Figure 2:**
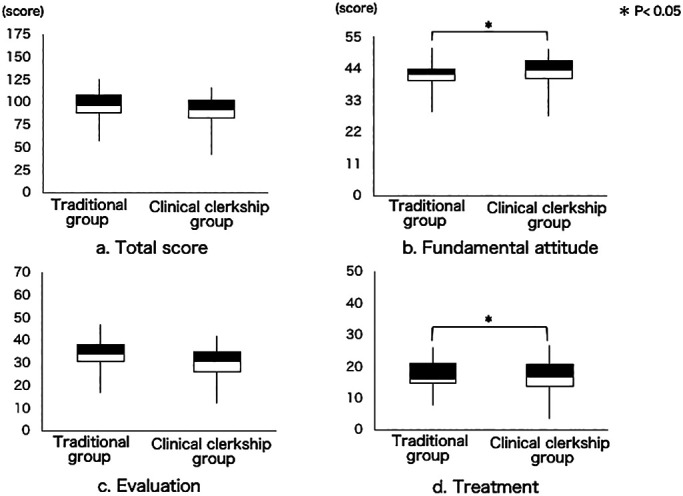
Comparison of skill acquisition between the traditional and clinical clerkship groups (clinical training evaluation grades). a. Total score. The total score for all items did not significantly differ between two groups. b. Fundamental attitude. The total score for items on fundamental attitude was significantly higher in the clinical clerkship group compared with the traditional group. c. Evaluation. The total score for evaluation technique did not significantly differ between the two groups. d. Treatment. The total score for treatment technique was significantly higher in the clinical clerkship group compared with the traditional group.

Figure 2 shows the results for the analysis of the clinical training grades. The median total score (out of 175) was 96.0 in the traditional group and 91.0 in the clinical clerkship group (Figure 2a). The median total score for training evaluation of fundamental attitude (out of 55) was significantly higher in the clinical clerkship group (44.0) than in the traditional group (42.0) (Figure 2b). The median total score for training evaluation of evaluation technique (out of 75) was 34.0 in the traditional group and 33.0 in the clinical clerkship group (Figure 2c). Finally, the median total score for training evaluation of treatment technique (out of 50) was significantly higher in the clinical clerkship group (16.0) than in the traditional group (15.0) (Figure 2d).

## Discussion

Our study showed that the stress factor items on the Brief Job Stress Questionnaire (quantitative and qualitative burden scores) were significantly higher in the clinical clerkship group compared with the traditional group. Quantitative burden items indicate whether the amount of a task is a stress factor, and qualitative burden items indicate whether the difficulty of a task or concentration required to perform a task is a stress factor. We postulated that the elevated burden in the clinical clerkship group was due to the increased contact with patients experienced by these students, because they participated in all patient care for which their supervisor was responsible. Hioki et al.^11^ administered the Brief Job Stress Questionnaire to 316 physical and occupational therapists. They reported the mean score for quantitative burden was 8.3±1.9 in physical therapists and 8.8±1.8 in occupational therapists, and that for qualitative burden was 9.3±1.5 in physical therapists and 9.5±1.4 in occupational therapists. These scores were higher than the scores in both groups in the present study. Although our study found that the score for the clinical clerkship group was higher than the traditional group, it was still lower than results reported by Hioki et al.^11^ This suggests that students undergoing clinical clerkship have increased stress factors because they are more comprehensively involved in the therapist’s work.

The scores on items related to the extent of the control over a task were significantly lower in the clinical clerkship group than the traditional group. These items indicate whether the pace of performing a task or self-determination of task priority is a stress factor. Our results suggested that students who experienced clinical clerkship had greater stress related to not easily being able to make decisions on their own. In traditional clinical training, students are forced to think on their own from the outset, and were often not allowed to treat patients if their thoughts were disorganized. However, in clinical clerkship, the supervisor gives advice and support to students and adjusts the difficulty of the task as appropriate, thereby delineating specific tasks to the students. Our results reflected these characteristics of clinical clerkship.

In terms of stress responses, we found that scores for irritability, fatigue, depression, and physical ailment were significantly lower in the clinical clerkship group compared with the traditional group. Moreover, scores for vitality were significantly higher in the clinical clerkship group compared with the traditional group. This indicated that both mental and physical stress responses were lower in clinical clerkship than in traditional training. Hioki et al.^11^ reported mean depression scores of 9.9±3.1 in physical therapists and 10.7±3.5 in occupational therapists, and mean physical ailment scores of 17.9±4.6 in physical therapists and 19.5±5.9 in occupational therapists. Compared with these results, the mean score in our traditional group indicated a greater stress response. The lower stress response for these items in the clinical clerkship group indicated an important positive effect of clinical clerkship.

For the social support items, scores for supervisor support were significantly higher in the clinical clerkship group compared with the traditional group. Supervisor support indicates how much support the student perceived from their supervisor. Our results revealed that students who experienced clinical clerkship perceived more support from their instructor. This reflected a key feature of clinical clerkship where students participate in a stepwise process of “observing,” “imitating,” and “practicing with the supervisor.” This process means supervisors have many opportunities to instruct and provide support, and communication is naturally promoted between the supervisor and student.

Results for the Japanese version of the Athens Insomnia Scale showed the clinical clerkship group had significantly lower scores and a smaller percentage of those with a score of ≥6 (high probability of insomnia) compared with the traditional group. This may be because instruction in clinical clerkship was generally finished within the training time, and time within the training time was allotted to work on tasks; therefore, time spent studying at home decreased, leading to improved sleep status. Stress is known to suppress sleep,^14^ and mental stress and difficulties overcoming stress have been suggested as reasons for problems related to sleep.^15^ Therefore, the lower stress response (as measured by the Brief Job Stress Questionnaire) in the clinical clerkship group compared with the traditional group may partly explain their better sleep status. A previous study on sleep status (using the Japanese version of the Athens Insomnia Scale) by Okajima et al.^13^ involving 163 healthy adults and 477 individuals with chronic insomnia reported mean scores of 2.64±2.02 in healthy adults and 11.81±4.50 in those with chronic insomnia. In our study, the traditional and clinical clerkship groups had higher scores than the healthy adults in the previous study, but lower scores than those with chronic insomnia. This suggested that students were at risk for developing insomnia during clinical training. Lack of sleep decreases attention and working capacity, leading to increased accidents at work.^16^ Moreover, many reports have shown associations between insomnia and depression^17^ and lifestyle diseases such as hypertension and diabetes.^18^ This highlights the need to check the sleep status of clinical training students and further investigate appropriate instruction and training formats.

Regarding clinical training grades, the total score for fundamental attitude during the first session of third-year clinical training was significantly higher in the clinical clerkship group compared with the traditional group. This may have been attributable to factors in the “observing” stage of technique acquisition such as: increased communication between the supervisor and student, increased interaction with patients, increased experience in practicing the items on fundamental attitude, and increased instruction related to this practice. The total score for treatment technique items was significantly higher in the clinical clerkship group than the traditional group, which may have been related to differences in proceeding with technique acquisition. For example, in traditional clinical training, students experience a treatment technique after acquiring the evaluation technique, whereas in clinical clerkship, students experience relatively easy treatment techniques from an earlier stage. However, the scores for evaluation technique items tended to be lower in the clinical clerkship group, albeit without statistical significance. This may be attributable to bias, because students in clinical clerkship primarily experience technical skills that the supervisor performs for patients; therefore, not all evaluation techniques can be carried out equally.

We compared the stress status, sleep status, and skill acquisition between students who experienced clinical clerkship and students who experienced clinical training before the introduction of clinical clerkship. However, there were some limitations in this study. First, although we randomly selected participants for the clinical clerkship group, we did not account for confounders using methods such as matching with propensity score. Moreover, skill acquisition was only examined for a portion of clinical training, and we did not investigate the effects of clinical clerkship in all stages of training at our university. In a further study, it may be necessary to investigate the status of skill acquisition of students over the course of their entire clinical training. Furthermore, although we used the clinical training grades that were in use before the introduction of clinical clerkship training, it may have been better to validate the effects of clinical clerkship on skill acquisition by combining the Objective Structured Clinical Examination (OSCE)^19^ as an evaluation index.

The present study examined clinical clerkship, which is a new educational method, and the supervisors’ lack of experience in this training format might have affected the results. Nonetheless, this study demonstrated the educational effects of clinical clerkship on stress status, sleep status, and some skill acquisition. Further studies should consider combining the OSCE in examining skill acquisition over all stages of clinical training, and validating the effects of supervisor experience in clinical clerkship.

## Figures and Tables

**Table1:** Clinical Training Grading

Fundamental attitude	Item	
	I-1	Is appropriately dressed
	I-2	Is timely and keeps deadlines
	I-3	Uses polite words and is respectful towards others
	I-4	Appropriately communicates verbally and nonverbally with the patient
	I-5	Follows the instructor’s instructions and can clinically participate as an assistant
	I-6	Demonstrates ambition and curiosity regarding knowledge and technique
	I-7	Appropriately self-manages and self-evaluates
	I-8	Understands the role and work of physical/occupational therapist
	I-9	Understands the protection of privacy and how to handle personal information
	I-10	Manages patient health (vitals) with consideration for patient safety
	I-11	Manages equipment and performs infection countermeasures
Evaluation	Item	
	II-1	Asks patients questions (medical interview)
	II-2	Examines reflex
	II-3	Measures ROM
	II-4	Measures MMT
	II-5	Examines sensory examination
	II-6	Carries out physical measurements
	II-7	Performs motor function evaluation of hemiplegia(SIAS, Brunnstrom stage test)
	II-8	Performs higher brain function tests
	II-9	Analyzes posture and movement
	II-10	Assesses ADL (FIM, Barthel index)
	II-11	Other ( ) *N: Not attempted if none→
	II-1 to 11: Appropriately evaluates individual patient	
	II-12	Obtains information from other division and ascertains the whole clinical picture of the patient
	II-13	Lists the problems and explains the reasons
	II-14	Discusses the results obtained from the evaluation and sets goals
Treatment	Item	
	III-1	Guides/assists in maintaining a sitting position and movements in daily activities
	III-2	Guides/assists in maintaining a standing position, standing up, and transferring
	III-3	Guides/assists in self-care (i.e. changing clothes, using the restroom)
	III-4	Guides/assists in walking and other modes of movement
	III-5	Performs basic treatment (ROM training, muscle-strengthening training)
	III-6	Other ( ) *N: Not attempted if none→
	III-1 to 6: Provides appropriate treatment to individual patient	
	III-7	Proposes a treatment program in order to reach goals
	III-8	Carries out the planned treatment program
	III-9	Evaluates during treatment as appropriate (includes observation)
	III-10	Modifies the treatment plan during treatment as necessary

<Fundamental attitude> 5: Can accurately understand and act without instruction 4: Can accurately understand and act after one instruction 3: Can accurately understand and act with repeated instruction 2: Can moderately understand and act with repeated instruction 1: Cannot understand or act even with repeated instruction 0: Cannot be allowed to carry out item N: Not attempted <Evaluation and treatment> 5: Can accurately evaluate/treat without instruction 4: Can accurately evaluate/treat with one instruction 3: Can accurately evaluate/treat with supervision/instruction 2: Can moderately evaluate/treat with supervision/instruction 1: Cannot evaluate/treat even with supervision/instruction 0: Cannot be allowed to carry out item N: Not attempted

**Table2:** Comparison of stress status between traditional group and clinical clerkship group (MHLW Brief Job Stress Questionnaire)

Item (total score)		Traditional group			Clinical clerkship group			Statistical significance
		Median	Mean	SD	Median	Mean	SD	
Stress factors								
Psychological quantitative burden of task (12)	○	6	5.5	1.7	8	7.8	2.1	**
Psychological qualitative burden of task (12)	○	5	5.2	1.7	6	6.1	1.7	**
Subjective feeling of physical burden (4)	○	2	1.9	0.7	2	2	0.7	
Interpersonal stress (12)	○	8	7.8	1.4	8	8.2	1.2	
Environmental stress (4)	○	3.5	3.4	0.7	4	3.5	0.6	
Extent of control over task (12)	◉	8.5	8.3	1.8	7	7.1	1.8	**
Extent of using technical skills (4)	◉	3	3.1	0.7	3	2.9	0.7	
Suitability (4)	◉	2	2.3	0.7	2	2	0.7	
Rewarding (4)	◉	2	1.9	0.8	2	1.7	0.7	
Stress response								
Irritability (12)	○	6	5.9	2.3	4.5	4.9	1.8	*
Fatigue (12)	○	8	7.8	2.3	6	6.7	2.3	*
Anxiety (12)	○	7.5	7.5	2.8	6	6.5	1.9	
Depression (24)	○	13.5	13.9	4	11	11	3.4	**
Physical ailment (44)	○	21	22.1	6.4	17	18.5	5.4	**
Vitality (12)	◉	6	6.7	2.5	8	7.8	1.9	*
Social support								
Support from training instructor (12)	◉	3	3.1	0.7	8	7.3	1.8	**
Support from teacher (12)	◉	7.5	7.6	1.8	8	7.5	1.8	
Support from family and friends (12)	◉	4	4.6	1.9	5	4.8	1.8	

○: Greater value indicates higher stress level ◉: Smaller value indicates higher stress level * P<0.05 ** P<0.01

**Table3:** Comparison of sleep status between traditional group and clinical clerkship group (Japanese version of Athens Insomnia Scale/Comparison of median and mean)

	Median		Minimum	Maximum	Mean		SD
Traditional group	*［	5.5	1	24	*［	6.9	4.4
Clinical clerkship group		4	0	11		4.7	2.9

(score) * P<0.05
